# Enoxaparin, effective dosage for intensive care patients: double-blinded, randomised clinical trial

**DOI:** 10.1186/cc8924

**Published:** 2010-03-18

**Authors:** Sian Robinson, Aleksander Zincuk, Thomas Strøm, Torben Bjerregaard Larsen, Bjarne Rasmussen, Palle Toft

**Affiliations:** 1 Department of Anaesthesia and Intensive Care, Odense University Hospital (OUH), Sdr. Boulevard 29. Odense C, DK 5000, Denmark; 2 Department of Biochemistry, Pharmacology & Genetics and Centre for Thrombosis and Haemostasis, OUH, Sdr. Boulevard 29. Odense C, DK 5000, Denmark

## Abstract

**Introduction:**

Intensive care unit (ICU) patients are predisposed to thromboembolism. Routine prophylactic anticoagulation is widely recommended. Low-molecular-weight heparins, such as enoxaparin, are increasingly used because of predictable pharmacokinetics. This study aims to determine the subcutaneous (SC) dose of enoxaparin that would give the best anti-factor Xa levels in ICU patients.

**Methods:**

The 72 patients admitted to a mixed ICU at Odense University Hospital (OUH) in Denmark were randomised into four groups to receive 40, 50, 60, or 70 mg SC enoxaparin for a period of 24 hours. Anti-factor Xa activity (aFXa) was measured before, and at 4, 12, and 24 hours after administration. An AFXa level between 0.1 to 0.3 IU/ml was considered evidence of effective antithrombotic activity.

**Results:**

Median peak (4 hours after administration), aFXa levels increased significantly with an increase in enoxaparin dose, from 0.13 IU/ml at 40 mg, to 0.14 IU/ml at 50 mg, 0.27 IU/ml at 60 mg, and 0.29 IU/ml at 70 mg (*P *= 0.002). At 12 hours after administration, median aFXa levels were still within therapeutic range for those patients who received 60 mg (*P *= 0.02).

**Conclusions:**

Our study confirmed that a standard dose of 40 mg enoxaparin yielded subtherapeutic levels of aFXa in critically ill patients. Higher doses resulted in better peak aFXa levels, with a ceiling effect observed at 60 mg. The present study seems to suggest inadequate dosage as one of the possible mechanisms for the higher failure rate of enoxaparin in ICU patients.

**Trial Registration:**

ISRCTN03037804

## Introduction

Geerts et al. [1] determined the prevalence of deep vein thrombosis (DVT) in intensive care unit (ICU) patients not receiving prophylaxis to be in the range of 10-80%. The critically ill patient is especially predisposed to thromboembolism, possessing many inherent risk factors: cardiac failure, trauma, sepsis, cancer, increasing age, and obesity [1-5]. The acquisition of others, for example: respiratory support with decreased mobility and invasive monitoring, further tips the scale in favor of thrombosis during the ICU stay. Thus, these patients should undergo routine assessment for venous thromboembolism (VTE). The use of routine thromboprophylaxis will probably be justified in most [1,2].

Discerning DVT in critically ill patients is difficult [6]. The history and physical examination are often of little use, and thus, these patients are vulnerable to a delay in diagnosis. Low-molecular-weight heparins (LMWHs) are often used as a safe and effective means of prophylaxis [7-9] against VTE in medical and surgical patients. However, the efficacy of LMWHs in critically ill patients is less certain [10].

The antithrombotic activity of LMWHs is often determined by aFXa assay, despite several studies finding no direct correlation between aFXa activity and clinical outcome. Mayr [11] concluded that the European standard daily dose of 40 mg enoxaparin [12] was ineffective in ICU patients to achieve the recommended, albeit unproven, aFXa levels of 0.1 to 0.3 IU/ml. Thus, existing guidelines in other patient populations should not be directly applied to critical care patients without further study [2].

Most intensive care physicians widely acknowledge the need for evidence-based guidelines for antithromboembolic prophylaxis in this patient population and lament the dearth of research on VTE in critical care patients [4]. This study aims to establish the optimal dose of enoxaparin for ICU patients.

## Materials and methods

### Study population

The sample population consisted of 72 consecutive patients admitted to the ICU who were ≥ 18 years of age, with a minimum stay of >24 hours. Patients weighing <50 kg or >90 kg were excluded. Likewise, patients with bleeding diathesis, those in need of an operation within the time frame of the study, pregnant patients, and patients requiring continuous veno-venous hemofiltration were deemed ineligible. We recorded patient demographics such as age, sex, weight, height, and body mass index (BMI). The diagnosis on admission was also noted, and patients were classified according to standard ICU severity-of-illness scoring systems (APACHE II, Acute Physiology and Chronic Health Evaluation, and SAPS II, Simplified Acute Physiology Score) on the day of entry into the study.

### Study design

A prospective randomised double-blinded study was conducted at an 18-bed tertiary medicosurgical ICU. The patients were randomized into four groups/arms by sequentially numbered, sealed envelopes to receive one of the following SC doses of enoxaparin: 40, 50, 60, or 70 mg for a period of 24 hours. Patients, investigators, and all other personnel involved in the conduct of the study were blinded to individual treatment assignments. Attending physicians received envelopes with the assigned doses, which they then prescribed. The test doses were dispensed by the nurses. Attending physicians and nurses were therefore not blinded.

On the day of the study, blood samples were drawn from indwelling catheters immediately before, and at 4, 12, and 24 hours after the administration of enoxaparin to determine anti-factor Xa (aFXa) activity, antithrombin (AT), prothrombin time (PT), activated partial thromboplastin time (aPTT), thrombin-antithrombin complexes (TAT), fibrinogen, platelets, and D-dimer. Serum creatinine, as well as creatinine clearance, was determined for each patient. Patients were considered nonresponders if they showed no change in anti-factor Xa levels from baseline after administration of enoxaparin.

The study was performed in accordance with ethical principles set forth in the Declaration of Helsinki and with local regulations. The Committee for Good Clinical Practice at OUH approved and monitored the study. Written informed consent was obtained from each patient where possible, or otherwise from the closest family member.

The funding sources for this research had no role in the design, data collection, analysis, interpretation, or reporting of this study.

### Medication

Enoxaparin (Klexane; Sanofi-Aventis Denmark A/S, Hørsholm, Denmark) was available as prefilled single-dose syringes containing 20 mg and 40 mg. Nurses were instructed to titrate the dosage carefully to avoid inaccuracies.

### Assay methods

Samples for aFXa activity of heparin in plasma and TAT were stored at -80°C before analysis, whereas samples for all other hemostatic parameters were analyzed within 2 hours of collection. The frozen plasma samples were thawed and assayed in batches. Levels of aFXa activity were determined by using a validated chromogenic assay kit (COAMATIC Heparin; Chromogenix, Instrumentation Laboratory Company, Lexington, KE, USA) with the substrate S-2732, and the apparatus (STA-R Evolution; Diagnostica Stago, Asnières, France). The TAT complexes were determined by using an enzyme-immunoassay (Enzygnost TAT micro; Siemens, Marburg, Germany).

### Outcome measures

The primary end point was peak anti-factor Xa levels. Secondary outcomes included AT, PT, aPTT, TAT, fibrinogen, platelets, and D-dimer.

### Statistical analysis

**Figure 1:**
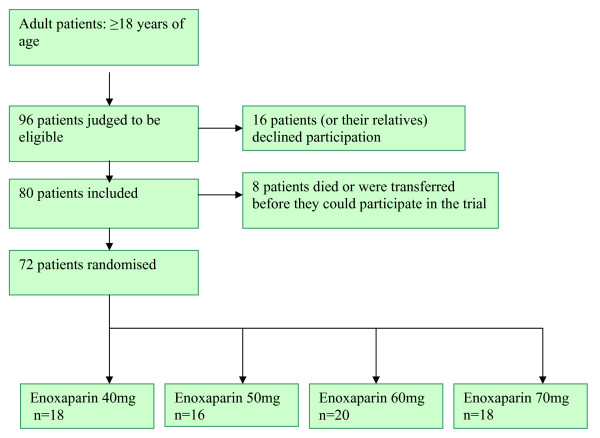
**Consort diagram**.

We calculated that 80 patients would be needed to detect an absolute difference of 30% in anti-factor Xa levels 4 hours after enoxaparin administration between the groups, assuming a power of 80% and a significance level of 5%. Statistical tests were two-sided and performed by using a significance level of 5%. A database was maintained in Microsoft Excel. The ICU patients were enrolled in the study between February 2006 and March 2009. The Consort diagram [13] shows patient disposition. (Figure 1).

### Patient characteristics

Thirty-two patients were admitted with pneumonia, exacerbation of chronic obstructive pulmonary disease (COPD), or other respiratory complaints. The next largest medical patient group, comprising 15 patients, consisted entirely of septic patients with or without the presence of shock. Among the surgical patients were nine admitted with ileus or other intestinal complaints, five with pancreatitis or infections in the biliary tree, and five with multiple trauma. At baseline, age, APACHE II score, BMI, and creatinine clearance did not differ significantly between the control group (the group receiving 40 mg enoxaparin), and the three test groups. SAPS II scores, however, were significantly lower in the group receiving 70 mg enoxaparin. All demographic data have been tabulated (Table 1).

**Table 1:** Clinical characteristics of the study population

Enoxaparin dose	40 mg (*n* = 18)	50 mg (*n* = 16)	60 mg (*n* = 20)	70 mg (*n* = 18)	*p*
Gender (m:f)	11:7	8:8	12:8	11:7	0.9

Age (years)	63 (57-71)	65 (55-75)	63 (55-70)	70 (46-74)	0.9

BMI (kg/m^2^)	26 (24-28)	24 (21-27)	26 (23-27)	25 (22-29)	0.5

SAPS II	37 (26-40)	38 (29-51)	38 (30-43)	29 (21-35)	0.04*

APACHE II	12 (9-15)	16 (11-19)	13 (9-15)	8 (6-15)	0.1

Medical:Surgical	13:5	11:5	16:4	11:7	

Creatinine clearance (ml/min)	8 2 (48-104)	58 (36-103)	65 (40-114)	57 (40-119)	0.8

BMI, Body Mass Index; SAPS II, Simplified Acute Physiology Score II; APACHE II, Acute Physiology and Chronic Health Evaluation. Data are expressed as median (25-75 percentiles) or number.

### Primary results

A strong positive correlation was found between the dose of enoxaparin and aFXa activity levels. Median peak (4 hours after administration) aFXa levels increased significantly with an increase in enoxaparin dose, from 0.13 IU/ml at 40 mg, to 0.14 IU/ml at 50 mg, 0.27 IU/ml at 60 mg, and 0.29 IU/ml at 70 mg (*P *= 0.002). 

**Figure 2:**
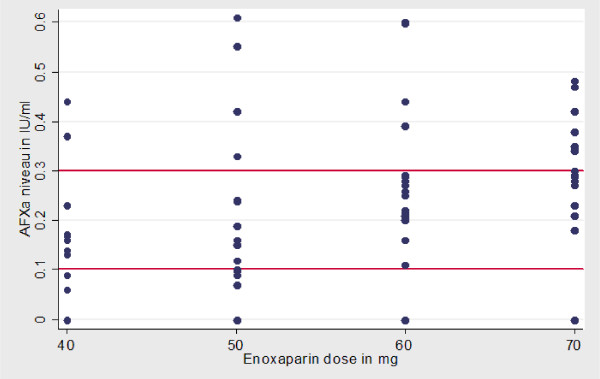
**Scatter diagram depicting anti-factor Xa levels for each dose, 4 hours after enoxaparin administration**. AFXa denotes anti-factor Xa.

**Figure 3:**
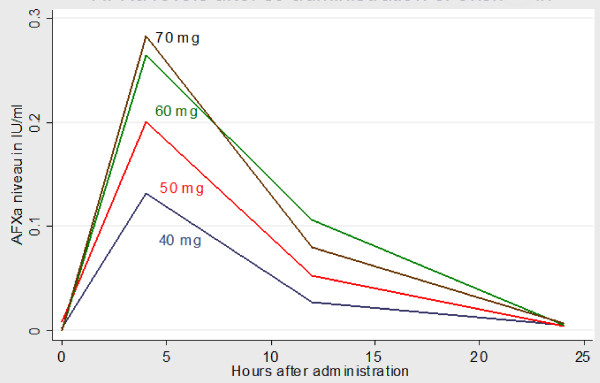
**Variation in anti-factor Xa over time for each dose of enoxaparin**. AFXa denotes anti-factor Xa.

The group receiving 40 mg had the highest number of non-responders (*n* = 5). The group receiving 70 mg had the highest number of patients with aFXa levels >0.3 IU/ml (*n* = 7). There was some variability in anti-factor Xa levels within the same dose group. For a dose of 40 mg enoxaparin, subtherapeutic levels occurred in 28% at 4 hours, 67% at 12 hours, and 83% at 24 hours. For a dose of 60 mg enoxaparin, subtherapeutic levels occurred in 5% at 4 hours, 30% at 12 hours, and 90% at 24 hours. At 12 hours after administration, median aFXa levels were still within therapeutic range for patients who received 60 mg (*P *= 0.02) (Figures 2 and 3).

### Secondary results

No significant difference was found in the PT, aPTT, AT, platelet count, fibrinogen, TAT complexes, or D-dimer levels between the four groups. No significant change from baseline was noted in these coagulation parameters within each group over the 24-hour study period (Table 2). We conducted a multiple regression analysis to determine whether creatinine clearance, BMI, age, or organ-failure scores had any influence on the levels of anti-factor Xa. No such influence was found.

**Table 2:** Other coagulation parameters at baseline

Parameter	Enoxaparin 40 mg	Enoxaparin 50 mg	Enoxaparin 60 mg	Enoxaparin 70 mg	*p*
TAT (μg/L)	8 (6-16)	10 (5-15)	13 (7-25)	11 (6-19)	0.5

aPTT (seconds)	41 (36-49)	44 (36-47)	40 (37-46)	43 (39-49)	0.9

PT (%)	73 (43-81)	73 (56-82)	60 (54-88)	67 (52-77)	1.0

Platelets (×10^9^/L)	211 (147-356)	220 (165-278)	201 (137-283)	299 (200-394)	0.4

AT (%)	77 (48-99)	72 (40-109)	72 (45-105)	82 (65-99)	0.9

Fibrinogen (μmol/L)	15 (13-18)	16 (12-18)	16 (13-19)	15 (11-21)	1.0

Ddimer (mg/L)	2 (2-5)	4 (2-5)	3 (2-4)	3 (2-5)	0.5

Data are expressed as median (25th-75th percentiles) or number.

### Adverse events

One minor nosebleed occurred in a patient with low platelet count (<65 × 10^9^/L) given 50 mg enoxaparin. A single death occurred in each of the 40 mg, 50 mg, and 60 mg groups. These patients did not die with/of bleeding.

## Discussion

Effectivity of enoxaparin is measured by aFXa activity. Peak concentration of aFXa activity occurs at 3 to 4 hours after SC enoxaparin injection [12,14]. AFXa activity levels between 0.1 and 0.3 IU/ml are considered to represent effective antithrombotic activity [11,15].

European standard dose of 40 mg enoxaparin SC, once daily, is often used as VTE prophylaxis in critical care patients [12]. Priglinger and Mayr et al. [10,11] demonstrated low effectiveness of this standard in achieving the recommended anticoagulant aFXa levels. This study concurs with the finding that 40 mg yielded subtherapeutic levels of aFXa in critically ill patients. The reasons for this apparent heparin resistance/failure are intriguing, and not entirely clear. Some authors suggest that physiologic mechanisms, or pharmacologic agents, for example, vasopressors, impair absorption of SC heparin through adrenergic-mediated vasoconstriction of peripheral blood vessels [11,14,16,17]. Others theorized that the presence of SC edema may decrease heparin absorption. However, no differences in aFXa activity after SC injection of 2,500 IU dalteparin for VTE prophylaxis between ICU patients with and without edema have been found [18]. Still others maintain that the presence of multiple organ dysfunction in ICU patients may alter drug metabolism, distribution, and binding to albumin and acute-phase proteins [11].

We included 80 patients in the trial (the same number of patients as the trial conducted by Mayr et al.); eight were transferred before they could participate. The remaining 72 patients were randomized and treated according to the intent-to-treat principle. We found a positive correlation between the dose of enoxaparin and aFXa levels ≤ 60 mg. Some variability in anti-factor Xa levels was found within the same dose group, suggesting a wide dispersion around the mean. In the study of Mayr et al. [11], ICU patients received 40 mg enoxaparin. Subsequent aFXa concentrations were below the therapeutic level in 27% of patients at 4 hours, 58% at 12 hours, and 88% at 24 hours. Those results reflect the findings in our study, in which, for those patients who received 40 mg enoxaparin, subtherapeutic levels occurred in 28% at 4 hours, 67% at 12 hours, and 83% at 24 hours. For a dose of 60 mg enoxaparin, subtherapeutic levels occurred in 5% at 4 hours, 30% at 12 hours, and 90% at 24 hours. A ceiling effect was observed at 60 mg, above which the potential for bleeding complications was introduced. Further clinical trials using 60 mg daily, with clinical end points, are needed.

No significant change from baseline occurred in aPTT, PT, AT, platelet count, D-dimer, and fibrinogen levels, irrespective of the dose of enoxaparin. These findings are consistent with other studies, which found no change in these hemostatic parameters with LMWH use [10,11]. TAT serves as an indicator of thrombin production and reflects the activation of coagulation. As enoxaparin moderates the conversion of prothrombin to thrombin, TAT levels might be expected to decrease with enoxaparin use [19,20]. In our study, a nonsignificant trend toward decreased TAT levels with increasing dose of enoxaparin was observed.

Mayr et al. [11] showed a significant negative correlation between aFXa levels and multiple organ dysfunction. Judging from the APACHE II score, the study patients all appear to be moderately to severely ill. The finding that patients who received 70 mg had lower SAPS II scores was unexpected, as the study was randomized; however, comparison of multiple parameters will invariably lead to one such parameter attaining significance purely by chance.

Periodic monitoring of aFXa levels is recommended in special populations (for example, in pregnant patients, children, patients with acute kidney injury (AKI), or in those at extremes of body weight [21]. The aFXa activity 3 hours after enoxaparin had a negative correlation with BMI [10], owing to reduced blood flow in fat tissues, resulting in decreased drug absorption [11], and thus obese patients were not included. Standard therapeutic-dose enoxaparin in patients with severe AKI led to accumulation, resulting in higher aFXa levels and a concurrent increased risk of bleeding complications [21,22]. Many advocate avoiding the use of LMWHs or using a lower dose of these agents combined with careful monitoring of drug levels and anticoagulant effects [1,23]. From the median creatinine clearance in Table 1, it is easily inferred that study patients had renal functions that ranged from being normal to only moderately impaired. This was done to prevent AKI from influencing the anticoagulant effect of the different enoxaparin doses.

One clear limitation of our study was that it was not designed to detect VTE. The patients had a drug exposure of only 24 hours and were not screened for VTE by using Doppler ultrasound. In addition, the nurses found it somewhat difficult to titrate such small doses of enoxaparin, and this may have resulted in the variability in anti-factor Xa levels seen within the same dose group. Despite this, the present study is both timely and relevant to issues facing intensive care physicians. The inclusion of 72 patients is quite good for a project of this nature, and the study was powered to 80%. In addition, our study generates the hypothesis of inadequate dosage being one of the possible mechanisms for the higher failure rate of enoxaparin in ICU patients.

Our findings cannot be extrapolated to mean that ICU patients receiving 60 mg are better protected than are those receiving a standard dose of 40 mg, as aFXa activity is only a surrogate parameter.

Most studies suggest that although aFXa activity cannot be directly related to clinical outcome [7,20,24,25], lower concentrations seem to result in less-effective prevention of VTE. One study has even demonstrated a statistically significant relation between aFXa levels and wound hematoma and thrombosis, with regression analysis suggesting that aFXa levels are predictive of outcome [15]. It remains to be established whether these results obtained with enoxaparin can be generalized to other LMWHs.

## Conclusions

Our study confirms that the standard dose of 40 mg enoxaparin yielded subtherapeutic levels of aFXa in critically ill patients. In the present study, 60 mg, or 1.5 times the standard dose, resulted in the best peak aFXa levels. The study seems to suggest inadequate dosage as one of the possible mechanisms for the higher failure rate of enoxaparin in ICU patients. Further trials using 60 mg daily, with clinical end points, are needed.

## Key messages

• The present study seems to suggest inadequate dosage as one of the possible mechanisms for the high failure rate of enoxaparin in ICU patients.

• 60 mg, or 1.5 times the standard dose, resulted in the best peak aFXa levels.

• Further trials using 60 mg daily, with clinical end points, are needed.

## Competing interests

The authors declare that they have no competing interests.

## Authors' contributions

PT conceived the research, included patients, reviewed the manuscript, and was responsible for the project's economy. SR included and followed up patients, interpreted the results, drafted the manuscript, and presented at conferences. AZ included and followed up patients and reviewed the manuscript. TS performed the statistical analysis and reviewed the manuscript. TBL participated in the design of the study and reviewed the manuscript. BR carried out the immunoassays. All authors read and approved the final manuscript.
